# Non-monotonic variation of the Kramers point band gap with increasing magnetic doping in BiTeI

**DOI:** 10.1038/s41598-021-02493-8

**Published:** 2021-12-02

**Authors:** A. M. Shikin, A. A. Rybkina, D. A. Estyunin, I. I. Klimovskikh, A. G. Rybkin, S. O. Filnov, A. V. Koroleva, E. V. Shevchenko, M. V. Likholetova, V. Yu. Voroshnin, A. E. Petukhov, K. A. Kokh, O. E. Tereshchenko, L. Petaccia, G. Di Santo, S. Kumar, A. Kimura, P. N. Skirdkov, K. A. Zvezdin, A. K. Zvezdin

**Affiliations:** 1 Saint Petersburg State University, Saint Petersburg, 198504 Russia; 2 Helmholtz-Zentrum Berlin für Materialien und Energie, BESSY II, 12489 Berlin, Germany; 3 Kemerovo State University, Kemerovo, 650000 Russia; 4 Sobolev Institute of Geology and Mineralogy SB RAS, Novosibirsk, 630090 Russia; 5 Novosibirsk State University, Novosibirsk, 630090 Russia; 6 A. V. Rzhanov Institute of Semiconductor Physics, Novosibirsk, 630090 Russia; 7 Elettra Sincrotrone Trieste, 34149 Trieste, Italy; 8 Hiroshima Synchrotron Radiation Center, Hiroshima University, Higashi-Hiroshima, Hiroshima 739-0046 Japan; 9 Graduate School of Science, Hiroshima University, Higashi-Hiroshima, 739-8526 Japan; 10 Prokhorov General Physics Institute of the Russian Academy of Sciences, Moscow, 119991 Russia; 11 Moscow Institute of Physics and Technology, Dolgoprudny, 141700 Russia; 12 P.N. Lebedev Physical Institute of Russian Academy of Sciences, Moscow, 119991 Russia

**Keywords:** Condensed-matter physics, Condensed-matter physics, Nanoscale materials

## Abstract

Polar Rashba-type semiconductor BiTeI doped with magnetic elements constitutes one of the most promising platforms for the future development of spintronics and quantum computing thanks to the combination of strong spin-orbit coupling and internal ferromagnetic ordering. The latter originates from magnetic impurities and is able to open an energy gap at the Kramers point (KP gap) of the Rashba bands. In the current work using angle-resolved photoemission spectroscopy (ARPES) we show that the KP gap depends non-monotonically on the doping level in case of V-doped BiTeI. We observe that the gap increases with V concentration until it reaches 3% and then starts to mitigate. Moreover, we find that the saturation magnetisation of samples under applied magnetic field studied by superconducting quantum interference device (SQUID) magnetometer has a similar behaviour with the doping level. Theoretical analysis shows that the non-monotonic behavior can be explained by the increase of antiferromagnetic coupled atoms of magnetic impurity above a certain doping level. This leads to the reduction of the total magnetic moment in the domains and thus to the mitigation of the KP gap as observed in the experiment. These findings provide further insight in the creation of internal magnetic ordering and consequent KP gap opening in magnetically-doped Rashba-type semiconductors.

## Introduction

Materials and systems that combine the properties of two-dimensional (2D) electron gas with strong spin-orbit coupling and internal ferromagnetism constitute one of the most promising platform for the future development of spintronics and quantum computing^1,2^. One of such material is the polar semiconductor BiTeI doped with magnetic metal atoms. Pure BiTeI has a layered crystal structure formed by the tripple-layer block (TLs) Te-Bi-I separated by van der Waals spacings^3–6^. Electronic structure of this material in the vicinity of the Fermi level is characterized by two parabolic-like energy bands, shifted along $k_{\Vert }$ from the $\Gamma$-point due to the Rashba effect ($E_{\pm }(k) \sim \frac{\hbar ^2}{2m^*}k^2\pm \alpha _R k$). The Rashba parameter ($\alpha _R$), which is proportional to the $k_{\Vert }$ shift, is one of the largest (3.8 eV Å) in BiTeI. As a result the parabolic bands are significantly spin split^3–8^. However, spin degeneracy remains only at the parabolas crossing point i.e. Kramers point (KP), since the time reversal symmetry is preserved. Doping of BiTeI with magnetic atoms leads to the lifting of the spin degeneracy and opening of the band gap at the KP (KP gap). The magnetization has to be directed perpendicular to the sample surface to open the gap in all $k_{\Vert }$. The spin structure of the state becomes “hedgehog”-shape in the vicinity of the KP and the electronic structure can be tuned to have one circular Fermi contour^9^. Similar spin degeneracy lifting can be observed in magnetic topological insulators for the topological surface state^10–15^ or Rashba-like state^16^.

Systems such as BiTeI with magnetic doping can be used as a basis for highly efficient generation and control of spin currents^2,17–22^. Therefore it can be used as spin filters, high-speed spin switches of memory elements, spin-Hall current devices etc. Moreover, the states with opposite momenta and thus spin orientation can be coupled through the proximity effect with a superconductor. This can lead to the formation of a topologically nontrivial superconducting phase^1^ and consequently, to the emergence of Majorana fermion modes with zero energy^23,24^. The above-mentioned effects in magnetically-doped BiTeI may significantly advance the technology of quantum computing and their solid-state implementation^1,25–27^.

In earlier works, devoted to experimental and theoretical study of V-doped BiTeI^9,28^, it was shown that the KP gap reaches the value of ~ 90 meV with the V concentration of 2%. Remarkably, the ab-initio calculations reveal KP gap no greater than 34 meV in this case^9^. This discrepancy could be related to unaccounted Bi-vacancies in the vicinity of V impurities, which can affect the magnetic moments of the nearest Te atoms and increase the gap value. KP gap up to 100 meV was also observed in ferroelectric semiconductor GeTe doped with Mn atoms up to 15% concentration which becomes a Rahsba-Zeeman system below the Curie temperature^29,30^. However, many features of the KP gap opening in Rashba systems are still weakly studied.

In the present work we study V and Mn-doped BiTeI samples with impurity concentrations of 0.5%, 2%, 3%, 6% for V and 2.5% for Mn by ARPES, scanning tunneling microscopy (STM), atomic force microscopy (AFM) and SQUID magnetometry. STM studies confirm the homogeneity and the concentration of magnetic impurities over the sample surface. From the analysis of the electronic structure we experimentally show that the KP gap value behaves non-monotonically with increase of doping level. Indeed, initially the gap increases up to 130–135 meV at 3% of V impurity. Then the gap saturates and reduces with further increase of the V concentration to 6%. The theoretical estimations show that such non-monotonic variation of the KP gap size is related to the appearance of antiferromagnetically coupled magnetic impurity (V, Mn) atoms above 3% of doping level in BiTeI. Consequently, the effective magnetic moment at each domain can be reduced, which is accompanied by the decrease in the KP gap value.

## Experimental results and discussion

**Figure 1:**
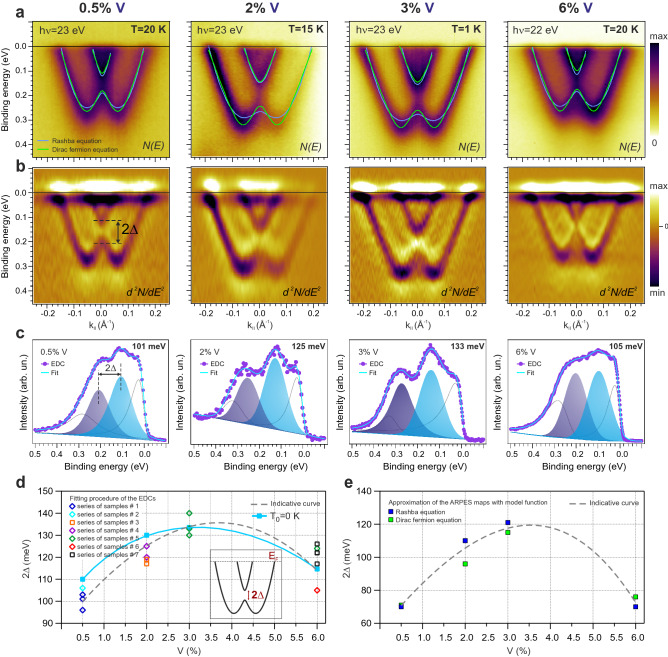
(**a**) ARPES dispersion relations N(E) for V-doped BiTeI with different concentration of V: 0.5% (T = 20 K), 2% (T = 15 K), 3% (T = 1 K) and 6% (T = 20 K) and their the second derivative $d^2 N / d E^2$ in (**b**). Approximations of the experimental Rashba bands with two model functions: Rashba equation (Eq. 1, blue curves) and Dirac fermion equation (Eq. 2, green curves) in (**a**). (**c**) The EDCs cut at the $\Gamma$ point ($k_{\parallel } = 0$ Å$^{-1}$) with the decomposition into spectral components. The components corresponding to the Rashba parabolas states at the KP gap is shown in blue and purple color. Dependence of the KP gap value ($2\Delta$) on V concentration (0.5, 2, 3 and 6 %) measured for the series of samples (see details in “Methods” section) derived from the EDC analysis (**d**) and model functions approximation (**e**). The dash lines show the non-monotonic dependence of the KP gap averaged over all measurements for each V concentrations as guide for the eye in (**d**,**e**). The schematic presentation of the measured parameter, namely the KP gap size 2$\Delta$, is shown in inset of (**d**). Solid blue line in (**d**) shows the KP gap size dependence at T = 0 K, estimated under the assumption of the surface Curie temperature of about 130 K.

Figure 1a shows the ARPES dispersion maps measured for V-doped BiTeI at different V concentrations: 0.5% V, 2% V, 3% V and 6% V. They are accompanied with the second derivatives ($d^2N/dE^2$) representation in Fig. 1b for better visualization. For a more precise and quantitative information about the KP gap size variation we analyze the energy distribution curves (EDCs) at the $\Gamma$-point ($k_{\Vert } = 0$ Å$^{-1}$) which are presented in (c). Here, the decomposition into spectral components is also reported. Blue and purple solid peaks locate the energy positions of the states at the edges of the KP gap. One can see that the KP gap value increases from $\sim$ 100 meV up to 130–135 meV with the V concentration rising from 0.5% to 3%, respectively. However, further increase in the V-concentration to 6% leads to a reduction of the KP gap down to 105 meV. The overall variation of the KP gap ($2\Delta$) for all measured spectra vs V concentration is presented in Fig. 1d. Here, we should note that the spectra were carried out at different temperatures in between 1 and 20 K. In order to compare the results of the KP gap at equal temperatures we correct the values with the assumption of their power law dependence on temperature (for the analysis see section 2 in Suppl. Mat.). Corrected values of the KP gaps for the results obtained from Fig. 1a at T = 0 K are presented in Fig. 1d by blue symbols and schematically connected for better visualization. As one can see the curve slightly changes, though, retaining non-monotonic behaviour. Experimental error of the KP gap estimation is less than 15–25 meV (depending on the ARPES setup; see “Methods” section) and it is mainly caused by angular broadening (see Ref.^12^). Measurements of several samples decrease total error and more accurately demonstrate the observed non-monotonic KP gap behaviour. Moreover measured samples were grown separately and tested on different ARPES stations giving similar results of the KP gaps. This reduces the possibility of accidental disturbances in the samples stoichiometry.

For the analysis of the KP gap dependence we use also a different approach based on the experimentally derived shape of the Rashba bands to distinguish the KP gap value. Initially, we carefully track the position of the bands for all measured spectra using the second derivative along energy (for horizontal features) and momentum (for vertical features) directions. We also improve our estimations by analysing 2D curvature plots of N(E)^31^. In order to derive the parameters of the Rashba bands we approximate experimental data with the massive Rashba equation:

1 $$\begin{aligned} E_{\pm }(k)= E_0 + \frac{\hbar ^2}{2m}k^2\pm \sqrt{\Delta ^2 + \alpha _R^2 k^2}, \end{aligned}$$

where $E_0$ is the band bottom, *m* is the effective mass, $\alpha _R$ is the Rashba parameter, and $2\Delta$ is the momentum-independent KP gap. These approximations are shown by blue curves in Fig. 1a. The resulting KP gap values are $2\Delta =70$ meV for 0.5%V, $2\Delta =110$ meV for 2%V, $2\Delta =120$ meV for 3%V, and $2\Delta =70$ meV for 6%V. They well fit the observed non-monotonic gap dependence (Fig. 1e) being, though, slightly lower than the KP gaps derived from the EDC analysis (Fig. 1d).

At a closer look, the Rashba equation does not fit the whole doped BiTeI band perfectly (see blue curves in Fig. 1a). Inconsistencies appear at the band’s bottom (see Fig. 1a). Indeed, it was realized that the band dispersion of IV–VI semiconductors can be better reproduced with a massive Dirac fermion model. Taken from Ref.^29^ the energy dispersion has the following shape:

2 $$\begin{aligned} E_\pm (k)=E_0-\sqrt{m^2_D v^4+\hbar ^2 v^2 k^2 \pm \frac{1}{4}\sqrt{\Delta ^4_D+16\alpha ^4_D k^2}} \end{aligned}$$

Here $m_D$ is the Dirac mass and represents the curvature for small *k*-values, *v* is the band velocity and represents the steepness of the bands for larger *k*-values, and $E_0$ is a band offset. $\alpha _D$ is the Rashba-like parameter and the $\Delta _D$ energy term is proportional to $\Delta$ from the massive Rashba equation (i.e. $2\Delta =\sqrt{m^2_D v^4+\frac{1}{4}\Delta ^2_D}-\sqrt{m^2_D v^4-\frac{1}{4}\Delta ^2_D}$). The massive Dirac fermion model has indeed better consistency with the experimental bands (see green curves in Fig. 1a). Obtained KP gaps within this model are following $2\Delta =71$ meV for 0.5%V, $2\Delta =96$ meV for 2%V, $2\Delta =115$ meV for 3%V, and $2\Delta =76$ meV for 6%V. Anyway, both models deliver close results for KP gaps.

One can also notice that the position of the Rashba bands, taken as the binding energy of the band minimum ($\text {E}_{{min}}$), is not constant for all measured spectra in Fig. 1a,b. While V-concentration increases till 3%, the Rashba band positions shift to higher binding energies (i.e. $\text {E}_{{min}}$ increases) and reach their maximum shift of about 60 meV. Further increase of V concentration leads to the backward move of the bands. One can find correlation of such behavior with the one observed for the KP gap (Fig. 1d,e). However, the KP gap size depends on V concentration, while the Rashba bands shift can be also related to differences in temperature or experimental environment.

**Figure 2:**
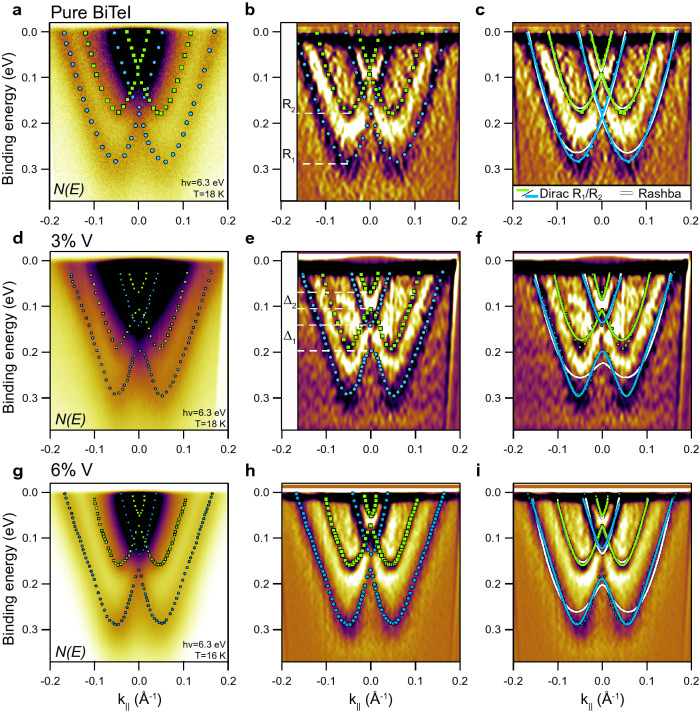
(**a**), (**d**), (**g**) ARPES dispersion relations *N(E)* measured with laser photoexitation (hv = 6.3 eV) for pure BiTeI and for 3% V and 6% V doped BiTeI. (**b**), (**e**), (**h**) EDC curvature plots of corresponding *N(E)* spectra with defined position of the first and second order Rashba states ($\text {R}_1$—blue circles and $\text {R}_2$—green squares). $\Delta _1$ and $\Delta _2$ are KP gaps in the $\text {R}_1$ and $\text {R}_2$ states. (**c**), (**f**), (**i**) Approximation of the experimental points with two model functions: Rashba equation (white curves for both $\text {R}_1$ and $\text {R}_2$) and Dirac fermion equation (blue and green for $\text {R}_1$ and $\text {R}_2$ respectively). The fitting derived KP gaps for 3% V (**f**) with the Rashba model are $\Delta ^R_1=78$ meV, $\Delta ^R_2=25$ meV and with the Dirac model are $\Delta ^D_1=62$ meV, $\Delta ^D_2=28$ meV. The KP gaps for 6% V (i) are $\Delta ^R_1=68$ meV, $\Delta ^R_2=20$ meV and $\Delta ^D_1=66$ meV, $\Delta ^D_2=24$ meV.

We have also studied doped BiTeI samples with 3%V and 6%V as well as pure BiTeI using micro ($\mu$)-spot laser based ARPES (hv = 6.3 eV). Corresponding dispersion relations accompanied with their EDC curvature plots^31^ are presented in Fig. 2. Laser-ARPES is known to have better spatial, momentum and energy resolution than regular synchrotron based ARPES^32^ (see “Methods” section). Moreover, misalignment from the normal emission (i.e. the $\Gamma$-point) is less significant at lower energy, which benefits for the precise KP gap estimation. Nevertheless, mean free path of ejected electrons rapidly rises at low photon energy and so additional bulk bands appear in the spectra such as Rashba bands from the next TLs. In Fig. 2a,d,g one can find the second order Rashba band (marked with green squares—$\text {R}_2$) additionally to the main one (marked with blue circles—$\text {R}_1$). $\text {R}_2$ is visually shifted to the lower binding energy for about 105 meV (3%V) and 130 meV (6%V) due to weaker band bending effect in the 2nd TL. Rashba bands from the next TLs are unresolved, but contribute to the high intensity region near the Fermi level. This region overlaps with the KP of the $\text {R}_1$ and $\text {R}_2$ bands obstructing EDC analysis of the KP gap. Therefore, here we relay only on the matching of bands with model functions (i.e. gapped Rashba and massive Dirac fermion equations, Eqs. 1 and 2 respectively). These approximations are presented in Fig. 2c,f,i for both Rashba bands. The parabolic functions of the Rashba model (white curves) fit worse again to the experimental bands than the Dirac model. Though, the fitting discrepancy between the models is significantly less for $\text {R}_2$ than for $\text {R}_1$, and in case of 3%V spectrum, both approximation results almost coincide. We estimate the KP gaps from the fitting for both $\text {R}_1$ and $\text {R}_2$ bands for pristine and doped BiTeI. In case of BiTeI the KP gaps for both $\text {R}_1$ and $\text {R}_2$ bands and both Rashba and Dirac models differ no more than 10 meV, which can be taken as value for the KP gap experimental resolution. For 3%V doped BiTeI the Rashba model provides KP gaps of $\Delta ^R_1=78$ meV, $\Delta ^R_2=25$ meV while the Dirac model $\Delta ^D_1=62$ meV, $\Delta ^D_2=28$ meV. The KP gaps for 6%V doped BiTeI are $\Delta ^R_1=68$ meV, $\Delta ^R_2=20$ meV and $\Delta ^D_1=66$ meV, $\Delta ^D_2=24$ meV. One can see that both models reveal close results. However, KP gaps for 3%V (62 and 78 meV) and for 6%V (66 and 68 meV) do not differ significantly and demonstrate rather saturation of the KP gap value than decrease. Still it can correlate with synchrotron-derived results, which on average also show saturation-like behaviour between 3 and 6%V in Fig. 1d for some spectra.

**Figure 3:**
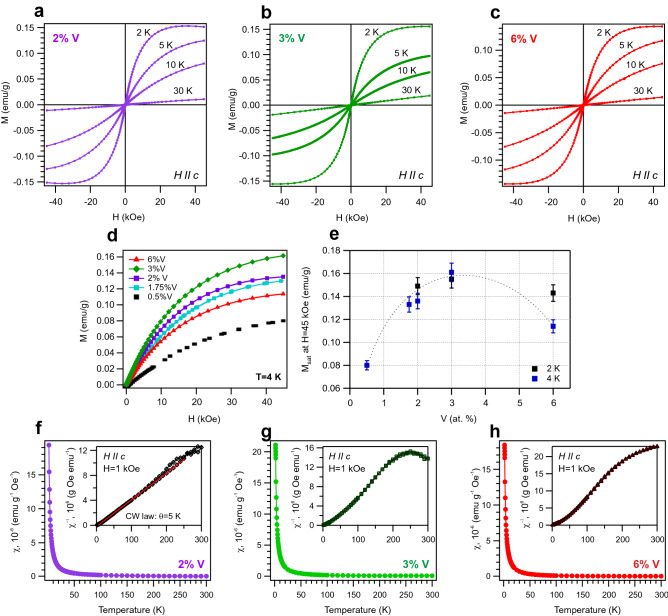
Comparative magnetization measurements using SQUID magnetometry. (**a**–**c**) Isothermal magnetization curves *M(H)* at several temperatures for 2%V, 3%V and 6%V-doped BiTeI, respectively. The external magnetic field was applied perpendicular to the surface plane, i.e. along *c*-axis. (**d**) Comparative analysis of the magnetization curves *M(H)* measured for 0.5%V, 1.75%V, 2%V, 3%V and 6%V-doped BiTeI at 4 K. (**e**) Dependence of the maximum *M* value at high magnetic field (saturation magnetization at H = 45 kOe) vs V-concentration. The dash line for the 4 K measurements is a guide for the eye. (**f**–**h**) Zero-field cooled (ZFC) and field cooled (FC) magnetic susceptibility curves ($\chi$) and inverse magnetic susceptibility ($\chi ^{-1}$) as a function of temperature for V-concentrations of 2%, 3% and 6% with 1 kOe field applied perpendicular to the surface plane.

The magnetic properties of the V-doped BiTeI and their possible modulation at different V-concentrations have been investigated using SQUID magnetometry. Figure 3a–c shows the isothermal magnetization curves *M(H)* for 2, 3 and 6% of V concentrations measured at temperatures from 2 to 30 K. The external magnetic field was applied perpendicular to the surface plane i.e. along the *c* axis. In order to compare the magnetization curves for various V concentrations Fig. 3d shows the series of *M(H)* curves at 4 K for 0.5%V, 1.75%V, 2%V, 3%V and 6%V. Figure 3e demonstrates the variation of the maximum value of magnetic moment from *M(H)* curves attainable at high magnetic field, which corresponds to the saturation magnetization ${M}_{{sat}}$ in the measured range of magnetic field. One can clearly see that the magnetic moment tends to rising when the V doping level increases up to 3%. Instead, as the V doping level goes from 3% to 6%, the magnetic moment slightly decreases. This tendency is observed for separate experiments at 2 K and 4 K. Such behavior correlates with the non-monotonic dependence of the KP gap size vs V doping reported in Fig. 1.

To obtain the information about the temperature critical points and to extract valuable information in magnetic phase transitions the so-called Arrott plot, which consists in plotting $M^{1/\beta }$ against $\textit{H/M}^{1/\gamma }$, with *H* the applied magnetic field and *M* the magnetization, was used^33,34^. The modified Arrott plot analysis using the 3D-Heisenberg model for ferromagnets shows that the bulk Curie temperature is around 5 K (see Fig. 6S in Suppl. Mat.).

The temperature dependence of the magnetic susceptibility ($\chi$) and inverse magnetic susceptibility ($\chi ^{-1}$) for 2%V, 3%V and 6%V measured at applied magnetic field of 1 kOe are presented in Fig. 3f–h. The diamagnetic background was subtracted by a linear fit at the high-temperatures data points. Zero-field cooled (ZFC) and field cooled (FC) magnetic susceptibility curves coincide at all temperatures. The estimation of critical temperature by fitting the experimental data with the Curie-Weiss law $\chi (T)=C/(T-\theta )$ gives the Curie-Weiss temperature $\theta$ of 5 K for 2%V-doped BiTeI (Fig. 3f) and the Curie constant $C=2.33\times 10^{-5}$ emu K g$^{-1}$. The positive Curie-Weiss temperature $\theta$ = 5 K suggests a possible tendency toward ferromagnetic coupling in the bulk material below the critical temperature. The attempts of the Curie-Weiss law approximation to the experimental data for 3%V and 6%V doping lead rather to uncertain results of critical temperature due to the non-linear character of $\chi ^{-1}(T)$ from 2 to 300 K.

The effects of retention of the KP gap at temperatures above the bulk magnetic transition temperature are also observed for other systems, such as magnetically-doped and intrinsic magnetic TIs (see, for example, Refs.^12–14^ and^15,16,35^, respectively), as well as magnetic Rashba materials (such as $\text {Ge}_{1-x}\text {Mn}_x$Te^29,30^). Similar gap opening retention at high temperatures were also observed in earlier works devoted to study magnetically-doped BiTeI^9,28^. This is a common and complex problem which is now under intense debates. There are several attempts to solve the problem of unclosed KP/DP gap. Namely, (i) spin fluctuations and (ii) increased surface transition temperature^35,36^. Spin fluctuations can form in magnetic TIs and preserve the out-of-plane spin anisotropy even above Curie/Neel temperature. As was shown for $\text {MnBi}_2\text {Te}_4$ such fluctuations exist up to 50–60 K which is 2–3 times higher than the magnetic transition temperature in this material^35,36^. Concerning (ii), it means that the bulk magnetic measurements cannot be directly used for the analysis of the surface magnetic properties modification. It can be rather manifested by the KP or DP gap opening in surface-sensitive ARPES measurements of magnetically-doped Rashba system and TIs. At the same time, we should note that defects can additionally modify the gap value, like Bi-vacancies in the vicinity of V impurities in BiTeI, which can affect the magnetic moments of the nearest Te atoms and increase the gap value^9^.

**Figure 4:**
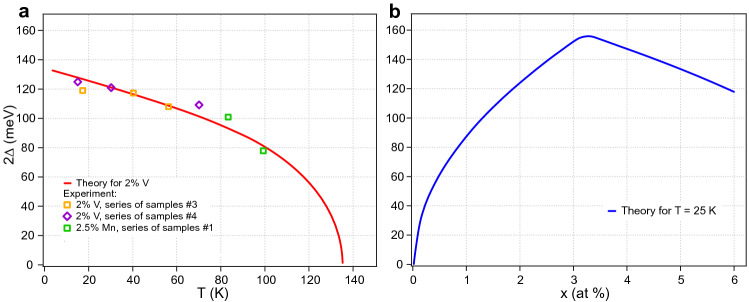
(**a**) Theoretical dependence of the energy gap $2\Delta$ on the temperature in comparison with experimental data for 2%V and 2.5%Mn doping. (**b**) Theoretical dependence of the energy gap on the concentration of magnetic impurities, taking into account possible pairing.

Our theoretical estimations provide a surface Curie temperature of about 130 K (see below). This value is significantly higher than the bulk transition temperature. However, with the above mentioned assumptions they may coexist. Moreover, the experimentally measured KP gap sizes at higher temperatures can testify to the high surface Curie temperature (see Fig. 4a and theoretical analysis below). At room temperature we observed the closing of the KP gap^9^. The analysis of the presented temperature-dependent variations in the experimental gap values in Fig. 1 for all concentrations show also a good agreement with the standard power law characteristic for the magnetic energy splitting $\Delta E=E_{0}(1-T/T_C)^{1/2}$ (with the critical temperature $T_C$ = 130 K), where $E_{0}$ is the energy gap at T = 0 K. The estimated values of the KP gap for different V-concentration at T = 0 K and at some different temperatures are shown in Table 1 of Suppl. Mat. The KP gap size dependence at T = 0 K, estimated under the assumption of the surface Curie temperature of about 130 K is shown in Fig. 1d by solid blue line and dots. All estimated temperature-dependent gaps correlate with the experimentally measured ones, including the values measured at temperatures between 1 and 100 K (see Suppl. Figs. 2S–4S, 5S and Fig. 4a). This may indicate the validity of the assumption about the enhanced surface Curie temperature (with the possible realization of spin fluctuations) and the corresponding dependence of the KP gap size on temperature measured by ARPES.

Noteworthy, the non-monotonic behaviour of the KP gap as in Fig. 1d,e was previously observed in Ref.^29^ for Mn-doped $\alpha$-GeTe. However, in this paper the gap starts to saturate at larger amount of Mn impurity ($\sim$ 10%) and shows no decrease under further Mn concentration rise (at least up to 15%). The saturation in the gap size with a further increase in Mn-concentration was associated in this work with Mn-phase segregation and corresponding antiferromagnetic coupling between neighboring Mn atoms that reduces the average FM moment per Mn atom.

Finally, we associate the decrease of the KP gap size above 3%V with a reduction in the effective magnetic moment caused by the formation and rise of AFM pair bonds. Actually, possible clustering of V somewhere in the sample volume instead of Bi substitution could reduce the V concentration influencing Rashba bands, which could also be followed by the reduction of the KP gap. To exclude this possibility we have additionally studied the V-doped BiTeI samples by STM and AFM in order to validate the absence of impurity clusters. Figure 1S in Suppl. Mat. shows the uniformity of the distribution of magnetic impurities and clearly demonstrates the absence of V-clusters for all studied V-concentrations. Below a theoretical analysis of the modulation of the magnetic properties of the system and the value of the KP gap depending on the level of doping with magnetic metals are presented.

## Theoretical analysis

To further investigate the physics of the observed phenomena, we have theoretically analyzed the changes in the gap at the Kramers point with respect to the concentration of the magnetic metal dopant and on the temperature.

Let us consider the interface of BiTeI doped by magnetic ions as the system with two dimensional (2D) electron gas with spin-orbit (Rashba) interaction and the local dopant magnetic moments. Under the assumption that ions do not interact directly with each other but only with conduction electrons the Hamiltonian can be written as:

3 $$\begin{aligned} {\hat{H}}={\hat{H}}_e+ J \sum \limits _{i=1}^{N} {\hat{S}}_i \cdot {\hat{s}}_i, \end{aligned}$$

where ${\hat{H}}_e$—Hamiltonian of the electron system, ${\hat{S}}_i$ and ${\hat{s}}_i$—spins of ions and electrons respectively, *N*—the number of ions and *J*—the exchange constant, which describes s-d interaction between free 2D Rashba electron gas and magnetic impurities system. Neglecting the fluctuations one can rewrite this expression in the following form:

4 $$\begin{aligned} {\hat{H}}={\hat{H}}_e + J \langle {\hat{S}}_i \rangle \sum \limits _{i=1}^{N} {\hat{s}}_i + J \langle {\hat{s}}_i \rangle \sum \limits _{i=1}^{N} {\hat{S}}_i - J N \langle {\hat{S}}_i \rangle \langle {\hat{s}}_i \rangle . \end{aligned}$$

In the mean field approximation one can obtain the decoupled Hamiltonian $H^{MF}=H^{MF}_e + H^{MF}_i - JNm_e^{'} M^{'}$, where $m_e^{'} = m_e / g \mu _B$, $M^{'} = M / g_i \mu _B$, $m_e$ and *M*—magnetizations of electron and ion subsystems respectively, $\mu _B$—Bohr magneton, *g* and $g_i$—g-factor of electron and ion respectively. The Hamiltonian of the ion subsystem has form $H^{MF}_i = Jm_e^{'} \sum \limits _{i=1}^{N} {\hat{S}}_i$, and the Hamiltonian of the electrons subsystem takes form:

5 $$\begin{aligned} H^{MF}_e = \sum \limits _{k} \psi _k^{+} \left( {\hat{H}}_e (k) + JM^{'} \frac{\sigma _z}{2} - \mu \right) \psi _k, \end{aligned}$$

where $\sigma _i$—Pauli matrices, $\mu$—chemical potential and ${\hat{H}}_e (k)$—one-electron Hamiltonian for BiTeI, which has the form:

6 $$\begin{aligned} {\hat{H}}_e(k) = \frac{\hslash ^2 k^2}{2m} {\hat{I}} + \alpha \left( \vec {k} \times \vec {z} \right) \vec {\sigma }, \end{aligned}$$

where *m*—effective mass of the electron, $\hslash$—Dirac constant and $\alpha$—the Rashba constant. Based on it, the electron energy can be written as:

7 $$\begin{aligned} E_e^{\pm } (k) = \frac{\hslash ^2 k^2}{2m} \pm \sqrt{\Delta ^2 + \alpha ^2 k^2} - \mu , \end{aligned}$$

where $\Delta = JM^{'}/2$. The energy gap observed in the experiment in this case corresponds to $2\Delta$.

Now let us consider two dimensional electron gas with this dispersion law. The mean energy (kinetic part) in this case can be written in form:

8 $$\begin{aligned} F(\Delta )=\sum \limits _{\vec {k} \sigma } E^{\sigma }_e (\vec {k}) {\hat{n}}_k= \sum \limits _{\sigma } \int \limits _{|\vec {k}| \le k_F} E^{\sigma }_e (\vec {k}) f(E-E_F) \frac{kdk}{2\pi }. \end{aligned}$$

Similarly, the number of electrons can be written as $n_e = \sum \limits _{\sigma } \int f(E-E_F) \frac{kdk}{2\pi }$. Then neglecting corrections with order $T^2$ (low temperature approximation) one can calculate the mean energy as:

9 $$\begin{aligned} F(\Delta )= & {} \frac{\hslash ^2}{16 \pi m}\left( k_{+}^4 + k_{-}^4 \right) + \frac{1}{6 \pi \alpha ^2} ( (\Delta ^2 + \alpha ^2 k_{+}^2)^{3/2} \\&- (\Delta ^2 + \alpha ^2 k_{-}^2)^{3/2} ) - \frac{E_F}{4 \pi } \left( k_{+}^2 + k_{-}^2 \right) , \end{aligned}$$

where $E_F$—Fermi level, $k_{\pm }$—the values of the wave vector given by the condition $E_e^{\pm } (k_{\pm }) = 0$. Using the expression for the numbers of electrons one can write $4\pi n_e = k_{+}^2 + k_{-}^2$. Since experiment shows that the Fermi level is much higher than the gap (at the Kramers point), for the definition of $k_{\pm }$ we use the parabolic dispersion law $E_e^{\pm } (k_{\pm }) \approx \frac{\hslash ^2 k_{\pm }^2}{2m} \pm \alpha k_{\pm } - E_F$. After simplifications, one can get:

10 $$\begin{aligned} F(\Delta )= const - \frac{m}{2\pi \hslash ^2}\Delta ^2. \end{aligned}$$

Then taking into account that $\Delta = \mu _B H$ one can write:

11 $$\begin{aligned} m_e = - \frac{\partial F(H)}{\partial H} = \chi H = \chi \frac{J}{2 g_i \mu _B^2}M, \end{aligned}$$

where $\chi = m \mu _B^2 / \pi \hslash ^2$. It is also worth noting that in our case we have an analogue of the Pauli paramagnetism of free electrons, which appears not in the external, but in the exchange field. Indeed, the susceptibility in our case can be rewritten as $\chi =\frac{n_e \mu _B^2}{(E_F + m \alpha ^2 / \hslash ^2)}$, while the Pauli susceptibility for 3D gas of free electrons has the form $\chi _P^{3D}=\frac{3 n_e \mu _B^2}{2 E_F}$.

Now, knowing the magnetization of the electron system, we can consider the magnetization of the ionic system. We will assume that the ions do not interact with each other directly and are in the effective exchange field created by electron subsystem (which clearly follows from the form $H^{MF}_i$). Then their magnetization can be described by Brillouin function as:

12 $$\begin{aligned} M = g_i \mu _B S_i N B_{S_i}\left( \frac{g_i \mu _B S_i H_{exch}}{T}\right) , \end{aligned}$$

where $H_{exch} = J \frac{m_e}{g g_i \mu _B^2}$. Using it and $m_e$ one can rewrite dopant subsystem magnetization:

13 $$\begin{aligned} M = g_i \mu _B S_i N B_{S_i}\left( \frac{\chi J^2 S_i}{2 g g_i \mu _B^3 T} M\right) . \end{aligned}$$

Considering the small magnetization *M* near the Curie temperature, we can expand the Brillouin function in a Taylor series. This gives the critical temperature in the form:

14 $$\begin{aligned} T_C = \frac{1}{6}\frac{\chi J^2}{g \mu _B^2} N S_i (S_i + 1). \end{aligned}$$

This relation will make it possible to find the value of the exchange constant *J* using experimental estimations of the critical temperature value $T_C$. After that, we can write down the dependence of the magnetization of ions in the region near $T_C$:

15 $$\begin{aligned} M = \frac{12 g g_i \mu _B^4 T}{J^3} \sqrt{\frac{5 g (T_C - T)}{N \chi ^3 S_i (S_i + 1)(2 S_i^2 + 2 S_i +1)}}. \end{aligned}$$

To consider the studied system not only near Curie temperature but also in the case of low temperatures instead of expanding the Brillouin function we can use the inverse Brillouin function in the form of a power series^37^. Using it, we can write the dependence T (M):

16 $$\begin{aligned} \frac{1}{T}= \sum \limits _{i=0}^{+\infty } b_{2i+1} \frac{2 g \mu _B^2}{N \chi J^2}\left( \frac{M}{g_i \mu _B N}\right) ^{2i}, \end{aligned}$$

where $b_{2i+1}$—coefficients of the inverse Brillouin function expansion with $S_i$ from paper^37^. Taking into account that $M = 2 \Delta g_i \mu _B / J$ one can rewrite the expression (16) as:

17 $$\begin{aligned} \frac{1}{T}= \sum \limits _{i=0}^{+\infty } b_{2i+1} \frac{2 g \mu _B^2}{N \chi J^2}\left( \frac{2\Delta }{JN}\right) ^{2i} \end{aligned}$$

and it can be used for the calculation of the temperature dependence of the energy gap ($2\Delta$).

For the numerical estimations of $2\Delta$ we consider V$^{2+}$ impurities, which have $g_i = 2$ and $S_i = 3/2$ due to orbital quenching, and the impurity concentration $N = 1.18 \times 10^{13}$ cm$^{-2}$ which correspond to 2%. We consider the first nine nonzero terms of the inverse Brillouin function expansion. The effective mass of the electron was estimated from ARPES experimental data as $m = 1.43 \times 10^{-28}$ g (i.e. $\approx 0.157\cdot m_0$, $m_0$—electron mass). Using these parameters we varied the $T_C$ value to find the best coincidence between the theoretical energy gap dependence on the temperature and the experimental data. This procedure lead us to critical temperature estimation $T_C \approx {130{-}135}$ K.

The theoretical dependence of the energy gap on temperature is shown in Fig. 4a together with the experimental points corresponding to the gap value obtained from the ARPES measurements, through the relative EDCs decomposition into spectral components presented in Figs. 2S–4S in Suppl. Mat., at various temperatures for 2%V and 2.5%Mn-doped BiTeI. One can see a slow decrease in the gap value with temperature for both types of magnetically-doped BiTeI with a tendency to close the gap at temperature above 130–140 K. And at room temperature the gap is closed as it was shown in Ref.^9^. From Fig. 4a the resulting theoretical dependence correlate very well with the experimental points.

Let us now consider the possibility of the formation of an antiferromagnetic pair by two impurities. Indeed, if two impurities are close enough then due to indirect exchange they will be oriented antiferromagnetically and therefore they will not contribute to the total magnetization.

We will assume that the size of the sample plate is $a\times b$, and the impurities interact if the distance between them is less than *r*. We will also assume that the first $N_0$ impurities do not interact with each other. The value $N_0$ can be estimated as $N_0 = ab\kappa / \pi r^2$, where $\kappa$ is effective parameter, responsible for the uniform filling of the plate with impurities and lying in the range from 0 to 1. Then, the probability of getting into the region inside a circle with radius *r* of one of the $N_0$ impurities for the ($N_0 + 1$)-th impurity is equal to $p = w N_0 \pi r^2 / ab$, where *w* is the degree of overlap of the circles with radius *r* of the first $N_0$ impurities. If we introduce a random variable $x_i$, which takes on the value 0 when an impurity finds itself in the region with radius *r* of other impurities and 1—when it does not, the mathematical expectation of the sum of such random variables $S_n = \sum _{i = N_0}^{N} x_i$ will be equal to the number of new unpaired impurities in addition to $N_0$. But according to the central limit theorem, it is equal to the number of impurities in excess of $N_0$ (denote $N_1$) multiplied by the expectation of a single random variable $\mu (x_i) = \sum p_i x_i = 1 - w N_0 \pi r^2 / ab$. It should be noted that here to apply the central limit theorem we neglect the interaction between impurities in excess of $N_0$, i.e. we consider that $N_0 \gg N_1$, and we consider that $N_ 1 \gg 1$. Then the real number of unpaired impurities can be estimated as:

18 $$\begin{aligned} N_{real} = N_0 + N_1 \left( 1 - \frac{w N_0 \pi r^2}{ab} \right) - N_1 \frac{w N_0 \pi r^2}{ab}. \end{aligned}$$

The last term here represents the number of impurities from $N_0$, which no longer contribute to magnetization due to pairing with some of the $N_1$ impurities. Taking into account that the total number of impurities is $N = N_0 + N_1$, one can get for $N > N_0$:

19 $$\begin{aligned} N_{real} = N (1 - 2 w \kappa ) + 2 w \kappa N_0. \end{aligned}$$

Then, under the condition $w \kappa > 0.5$, the real number of unpaired impurities begins to decrease after that the number of impurities exceeds $N_0$. In this case, $N_ {real}$ should be substituted in all formulas for calculating *M* and $2\Delta$ instead of *N*. For numerical estimations let us consider $w \kappa = 0.75$, $T = 25$ K so that the experimental estimation concentration related to $N_0$ is slightly about 3% V doping. The theoretical dependence of the energy gap on the number of impurities, taking into account possible pairing, is shown in Fig. 4b and shows the observed non-monotonic variation. Therefore, from these results the assumption of a possible antiferromagnetic pairing between the impurities leads to a behaviour similar to that observed experimentally, thus supporting the proposed scenario.

## Conclusion

We present experimental observation of non-monotonic dependence of the Kramers point band gap (KP gap) on magnetic dopant concentration in V-doped BiTeI using ARPES. The KP gap increases with V concentration up to 3% and reaches its maximum value of $130{-}135$ meV according to synchrotron-based ARPES. A further increase in V concentration leads to mitigation of the KP gap by $105{-}125$ meV depending on the sample. Estimations of the KP gap with the gapped Rashba and massive Dirac fermion models as well as measurements by means of laser-based ARPES also confirm the observed non-monotonic dependence. This can also be supported by the similar behaviour of the magnetization saturation value derived from SQUID measurements. The estimation of the critical temperature by the Curie-Weiss law and the modified Arrott plot method of the isothermal magnetization curves M(H) give the bulk Curie-Weiss temperature of about 5 K. While for the surface, the theoretical estimations provide a Curie temperature of about 130 K. This high surface Curie temperature is supported by measurements of the KP gap size at different temperatures. Theoretical analysis shows that the non-monotonic behavior can be explained by the increase of antiferromagnetic coupled atoms of magnetic impurities above a certain doping level. This leads to the reduction of the total magnetic moment at the domains and thus to the mitigation of the KP gap as observed in the experiment.

## Methods

High-quality V- and Mn-doped BiTeI single crystals with various concentrations were synthesized using Bridgman method in Novosibirsk State University. The doping of V (Mn)-impurities is given in atomic percentages where V (Mn) magnetic atoms mainly replace the Bi atoms. The level *x*% of V(Mn) doping corresponds to stoichimetry of $\text {Bi}_{(1-x/100)}$V(Mn)$_{(x/100)}$TeI. Clean sample surfaces were obtained by ultrahigh vacuum cleavage.

The measurements of ARPES dispersion maps were carried out at the BaDElPh beamline at Elettra (Trieste, Italy), the BL-9 beamline at HiSOR (Hiroshima, Japan), the One-cube end station and RGBL-2 end station at BESSY II (Helmholtz-Zentrum Berlin, Germany), spin-ARPES end station at MAX-Lab (Lund, Sweden) using synchrotron radiation with photon energy in the regions 20–25 eV and at the $\mu$-Laser ARPES system ($h \nu$ = 6.3 eV) at HiSOR (Hiroshima, Japan) with improved angle and energy resolution and a high space resolution of the laser beam (spot diameter around 5 μm). The spectra were measured using a Scienta R4000 or a Specs Phoibos 150 analyzer with an incidence angle of the laser and synchrotron radiation of 50$^\textsc {o}$ relative to the surface normal at temperatures between 1 and 20 K.

In Figs. 1d,e and 4 the series of ARPES measurements results denoted by colored points are presented. In Fig. 1d,e the Series #1 corresponds to the 0.5%V-doped BiTeI samples, measured at BESSY II; the Series #2 to 0.5%V-doped BiTeI measured at MAX-Lab; the Series #3 to 2%V-doped BiTeI measured at Elettra; the Series #4 to 2%V-doped BiTeI measured at HiSOR; the Series #5 to 3%V and 6%V-doped BiTeI measured at BESSY II; the Series #6, #7 to 6%V-doped BiTeI measured at Elettra. In Fig. 4 the sample Series #4 and #3 corresponds to the 2%V-doped BiTeI samples measured at HiSOR and Elettra, respectively. The sample Series #1 in Fig. 4 was measured for 2.5%Mn-doped BiTeI at MAX-Lab. The base pressure during all photoemission experiments was better than $1{-}2 \times 10^{-11}$ mbar.

The photoelectron spectra (EDCs) at $k_{\parallel }$ = 0 Å$^{-1}$ obtained from the ARPES dispersion maps were decomposed into components by fitting procedure for the analysis of the gap size at the Kramers point. We optimized the fit by two main components at the Kramers point, the asymmetric component near the Fermi level corresponding to the features of iodine electronic states, and the small component at higher binding energy due to the features of the valence band states. The line shape and the width of the two main components were defined by the fitting procedure of the parabolic branches. Raw EDC data are shown in Fig. 1 by circles along with the best-fit results, the corresponding components and the background.

Measurements of magnetic properties were carried out in the Resource Center “Center for Diagnostics of Materials for Medicine, Pharmacology and Nanoelectronics” of SPbU Research Park using a SQUID magnetometer with a helium cryostat manufactured by Quantum Design. The experiments were performed in a pull mode in terms of temperature and magnetic field. The magnetic field was applied perpendicular to the sample surface. STM images were obtained using a Omicron VT SPM in the Resource Center “Physical Methods of Surface Investigation” of SPbU Research park at room temperature. AFM measurements were carried out under atmospheric conditions and at room temperature in the tapping mode on a NT-MDT Solver Pro-M microscope at the SPbU laboratory.

## Supplementary Information

Supplementary Information.

## Data Availability

The authors declare that the data supporting the findings of this study are available within the paper and the Supplementary Information.
