# Supplementary material for: Non-monotonic variation of the Kramers point band gap with increasing magnetic doping in BiTeI
Source: Sci Rep. 2021 Dec 2;11:23332. doi: 10.1038/s41598-021-02493-8 (PMC8639783; doi:10.1038/s41598-021-02493-8)
Supplement: Supplementary file 1 — Supplementary Information. [file 41598_2021_2493_MOESM1_ESM.pdf]

Supplementary material for
Non-monotonic variation of the Kramers point band gap with increasing magnetic doping in BiTeI

A. M. Shikin[1,*], A. A. Rybkina[1], D. A. Estyunin[1], I. I. Klimovskikh[1], A. G. Rybkin[1], S. O. Filnov[1], A. V. Koroleva[1], E. V. Shevchenko[1], M. V. Likholetova[1], V. Yu. Voroshnin[1,2], A. E. Petukhov[1], K. A. Kokh[1,3,4], O. E. Tereshchenko[1,5,6], L. Petaccia[7], G. Di Santo[7], S. Kumar[8], A. Kimura[9], P. N. Skirdkov[10,11], K. A. Zvezdin[10,11], and A. K. Zvezdin[10,11]

[1]Saint Petersburg State University, Saint Petersburg, 198504, Russia
[2]Helmholtz-Zentrum Berlin für Materialien und Energie, BESSY II, D-12489 Berlin, Germany
[3]Kemerovo State University, 650000, Kemerovo, Russia
[4]Sobolev Institute of geology and mineralogy SB RAS, 630090, Novosibirsk, Russia
[5]Novosibirsk State University, Novosibirsk, 630090, Russia
[6]A. V. Rzhanov Institute of Semiconductor Physics, Novosibirsk, 630090, Russia
[7]Elettra Sincrotrone Trieste, Trieste, 34149, Italy
[8]Hiroshima Synchrotron Radiation Center, Hiroshima University, Higashi-Hiroshima, Hiroshima 739-0046, Japan
[9]Graduate School of Science, Hiroshima University, Higashi-Hiroshima, 739-8526, Japan
[10]Prokhorov General Physics Institute of the Russian Academy of Sciences, Moscow, 119991, Russia
[11]Moscow Institute of Physics and Technology, Dolgoprudny, 141700, Russia

*ashikin@inbox.ru

1. Characterization of the V-doped BiTeI surface by STM and AFM

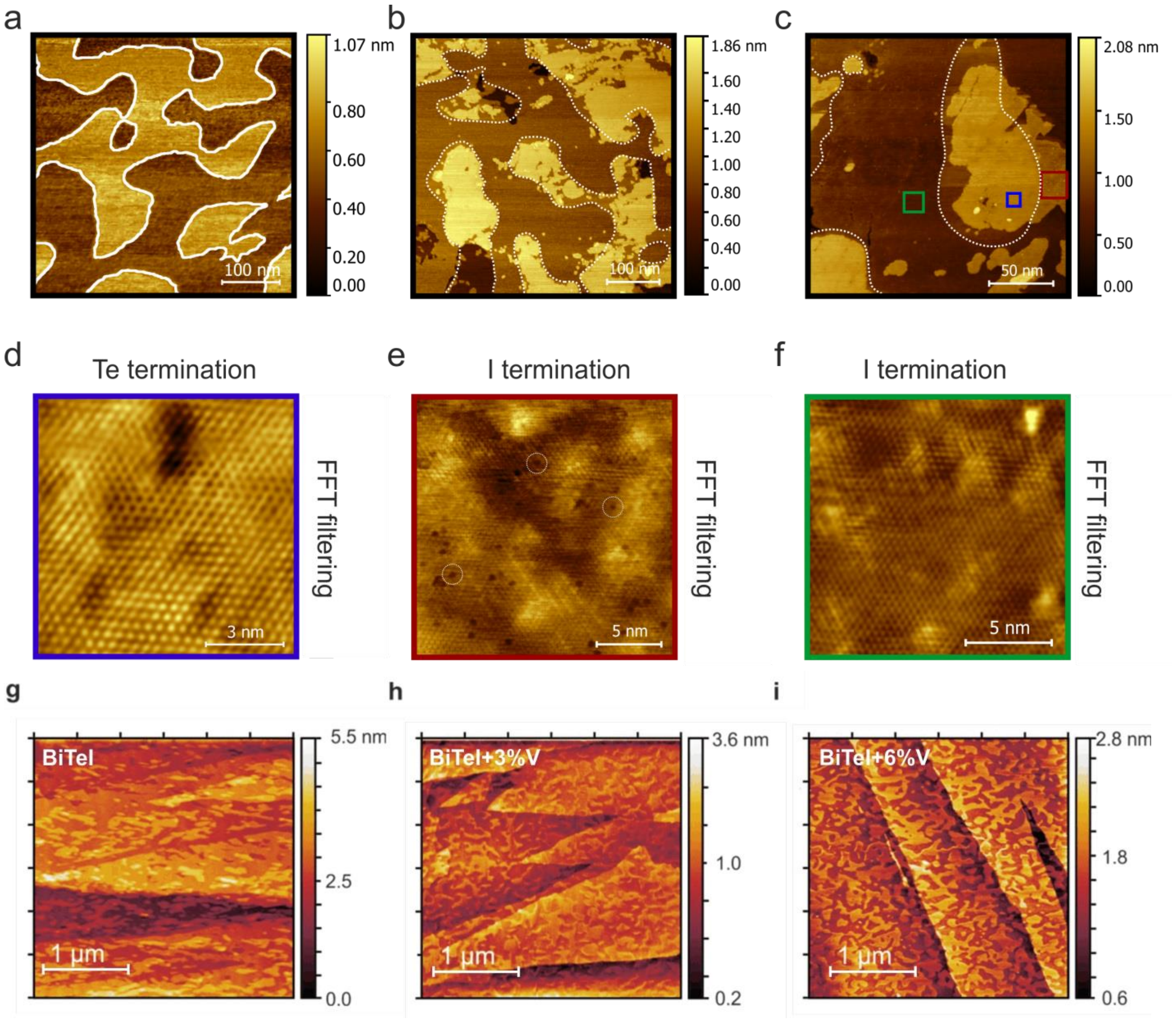

Fig. 1S. STM images of BiTeI with 3% concentration of V: large area images obtained at a bias voltage V_t = 0.5 V and a tunneling current I_t = 0.1 nA (a), V_t = 1.46 V, I_t = 0.01 nA (b) V_t = 1 V, I_t = 0.1 nA (c) with the colored squares where the atomically resolved STM images of Te and I surface terminations were obtained at V_t = 0.66 V and I_t = 0.3 nA (d), V_t = 1 V, I_t = 0.15 nA (e) and V_t = 1 V, I_t = 0.1 nA (f). STM data in panels (d)-(f) were analyzed and filtered with 2D Fast Fourier transform (FFT) for the noise reduction and the visibility gain of periodic features. (g)-(i) AFM images of pristine BiTeI, and BiTeI with 3% and 6% concentration of V, respectively.

Large area STM images show approximate 1:1 ratio of Te and I surface terminations (the border of the different terminations is shown by solid and dotted white lines in Figs. S1 (a-c)). It agrees well with previous STM/STS study of clean BiTeI surface [1]. Moreover, Te and I terminations, which are characterized by step height of about 1.8 Å, can be accompanied with an additional step height of about 7.2 Å (see, Figs. S1(b,c)) indicating single unit cell steps of BiTeI. Since the two possible surface terminations induce energy shifts of the relative electronic bands, the surface termination can be well identified by STS measurements (dI/dV curves) [1,2]. Figs. S1 (d-f) show the different terminations with atomic resolution, indicating a good quality of

our V doped samples. Panel (f) was measured at different tunnel current and scale then panel (e) to obtain better atomic resolution.

Defects bright and dark in color are often found at the topmost I termination, which can be associated with defects in the underlying Bi and Te layers [2]. We have revealed such atomic defects (marked by white circles in Fig. S1(e)) which are different in shape on the clean BiTeI surface but similar to the Ag substitution defects in Bi layer [2]. The defects density (approx. 1.5 defects per 10 nm^2) agrees with the density of Bi atoms substituted with V atoms for a 3% concentration of V with respect to Bi.

The AFM images measured in the tapping mode under atmospheric conditions reveal the presence of Te and I surface terminations for pure BiTeI and with 3% and 6% concentrations of V (Figs. S1 (g-i)). The surface of the samples is homogeneous on a large scale without formation of large V clusters.

[1] Butler, C., Yang, H., Hong, J. et al., Mapping polarization induced surface band bending on the Rashba semiconductor BiTeI, Nat. Commun. 5, 4066 (2014), https://doi.org/10.1038/ncomms5066
[2] Y. Kohsaka, M. Kanou, H. Takagi, T. Hanaguri, and T. Sasagawa, Imaging ambipolar two-dimensional carriers induced by the spontaneous electric polarization of a polar semiconductor BiTeI , Phys. Rev. B 91, 245312 (2015), https://doi.org/10.1103/PhysRevB.91.245312

2. Temperature dependent ARPES

Figs. 2S and 3S show ARPES dispersion maps and corresponding EDCs at the Γ-point measured for 2% V-doped BiTeI at different temperatures. The EDCs fitting procedure into spectral components as described in main article demonstrates the modification of the band gap at KP for 2% V doped BiTeI vs temperature for different series of samples and measurements. It can be seen from the presented ARPES dispersion maps in Fig. 2S that with an increase in temperature from 15 to 70 K, the KP band gap decreases approximately from 125 to 109 meV. At a temperature of 70 K, a shift of the Fermi level is observed in the measured spectra, which may be associated with the desorption of residual gases from the cryomanipulator as the temperature increases. Anyway, this does not change the structure of the measured dispersion dependencies. In fact, for the second series (Fig. 3S, and series of samples #3 in Fig. 4(a)) the gap decreases from 119 meV at 17 K to 108 meV at 56 K. However, in both cases the KP gap remains open in the measured temperature ranges, although with a tendency to decrease the gap value as the temperature increases.

Fig. 4S demonstrates an analogous variation in the ARPES dispersion maps and corresponding EDCs at the Γ-point (with decomposition into spectral components) measured for 2.5% Mn-doped BiTeI sample at temperatures between 83 and 99 K. The gap value is slowly decreasing with temperature also in this case, and remains open in this temperature range.

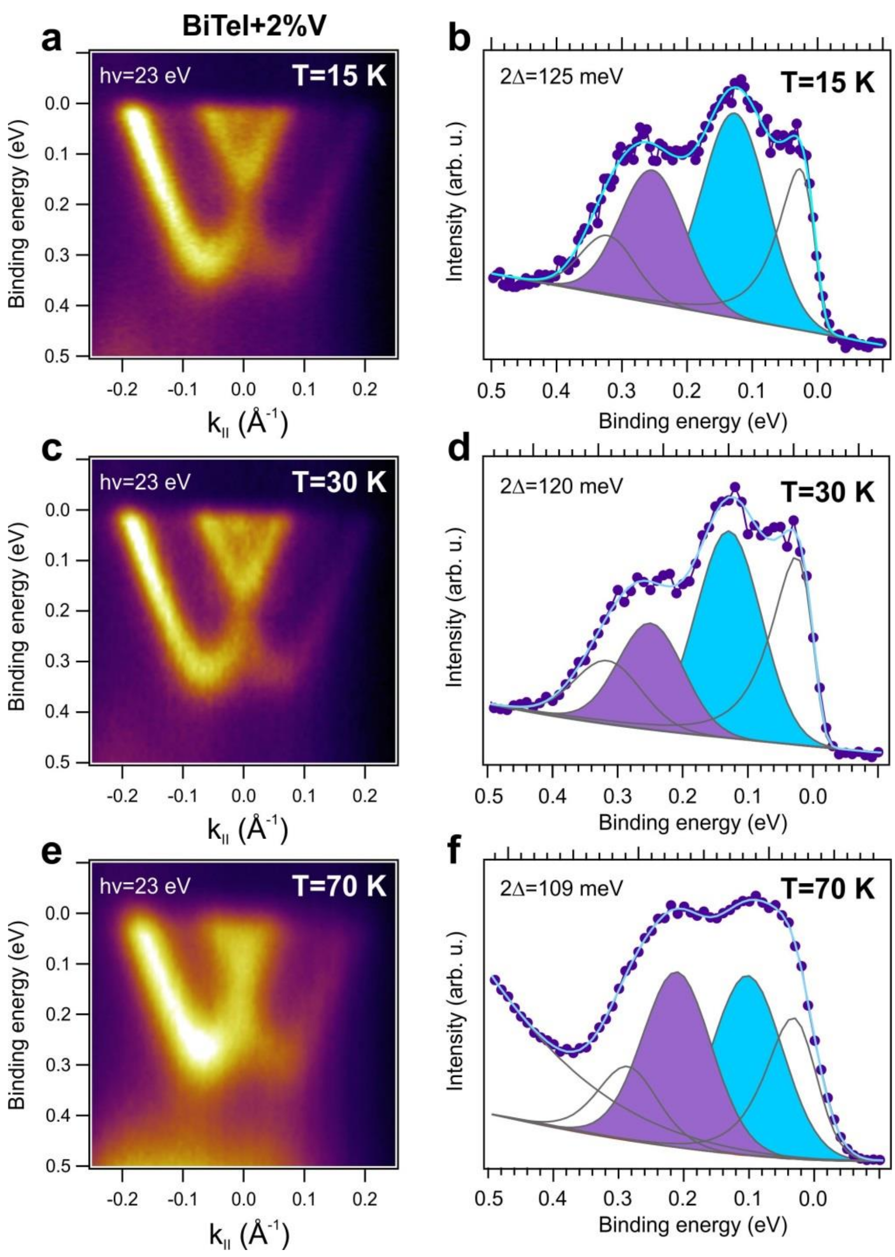

Fig. 2S. (a, c, e) – ARPES dispersion maps of 2% V-doped BiTeI measured at the indicated temperatures in the range 15-70 K. (b, d, f) — Corresponding EDCs measured at the Γ point (k_{II} = 0 Å^{-1}) with decomposition into spectral components.

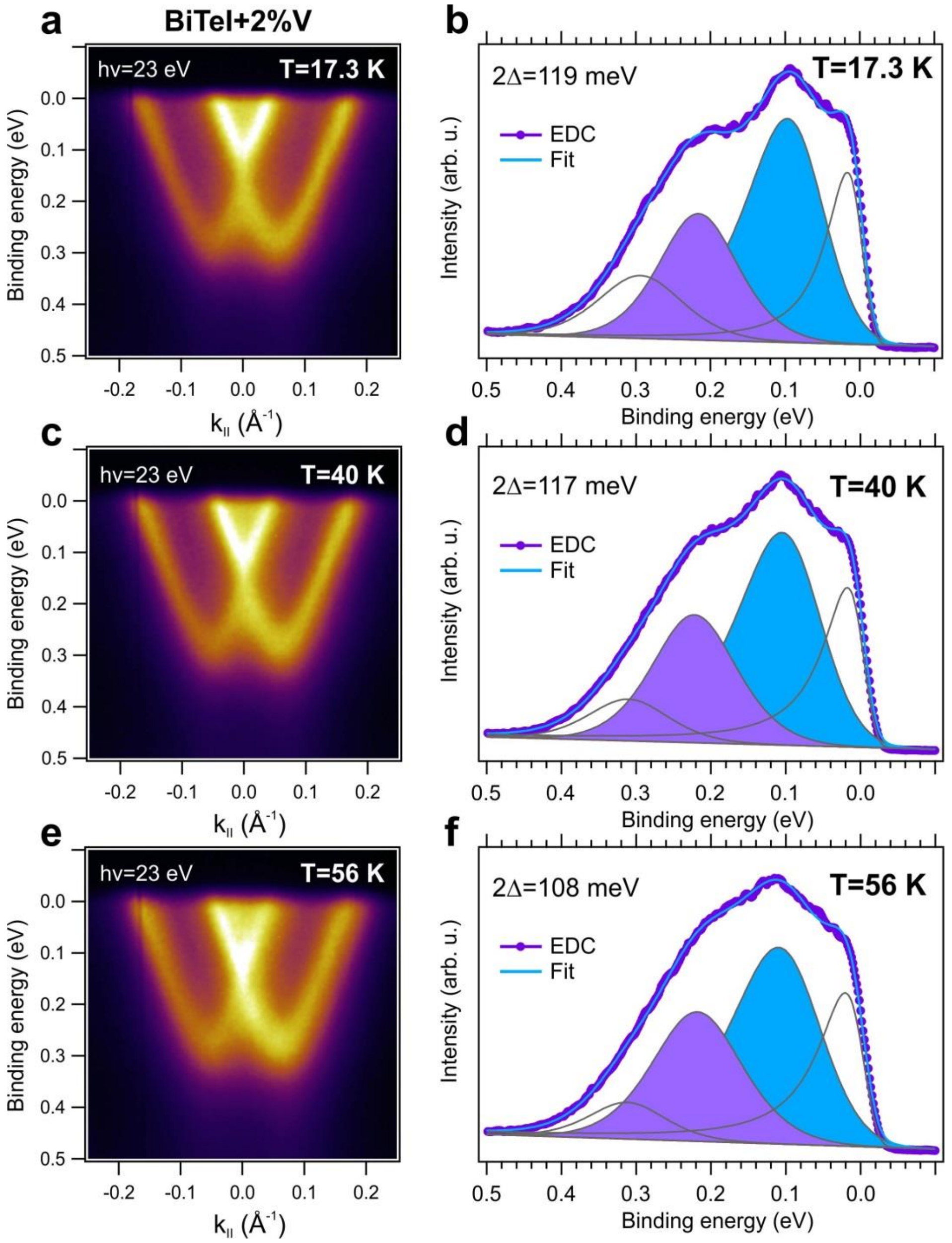

Fig. 3S. (a, c, e) — ARPES dispersion maps of 2% V-doped BiTeI measured in the indicated temperatures in the range 17 and 56 K. (b, d, f) — Corresponding EDCs measured at the Γ point (k_{II} = 0 Å^{-1}) with decomposition into spectral components.

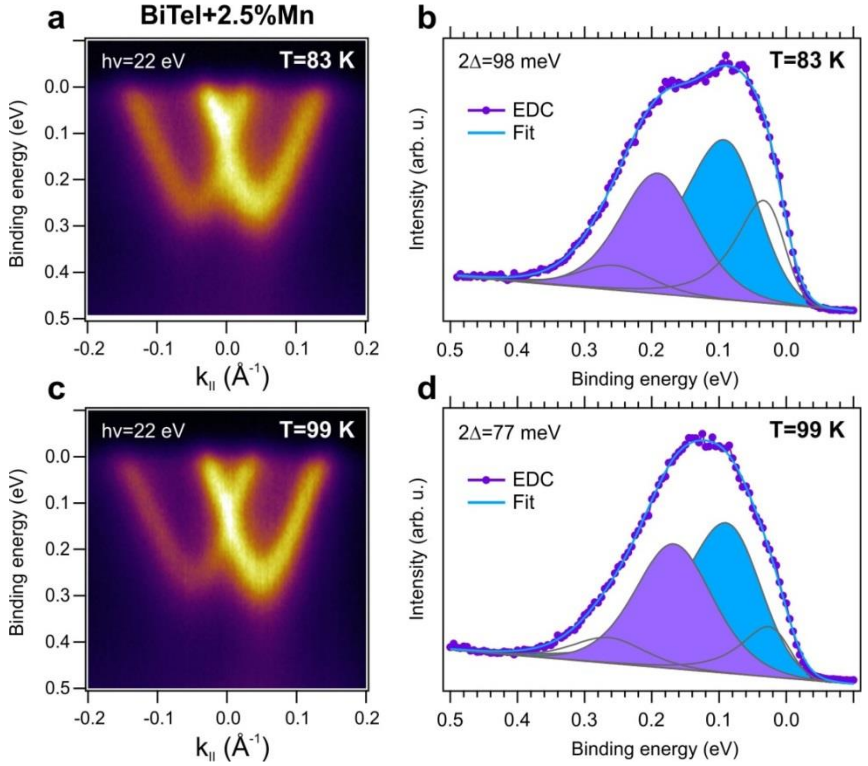

Fig. 4S. (a,c) – ARPES dispersion maps of 2.5% Mn-doped BiTeI measured at 83 and 99 K. (b,d) — Corresponding EDCs measured at the Γ point (k_{II} = 0 Å^{-1}) with decomposition into spectral components.

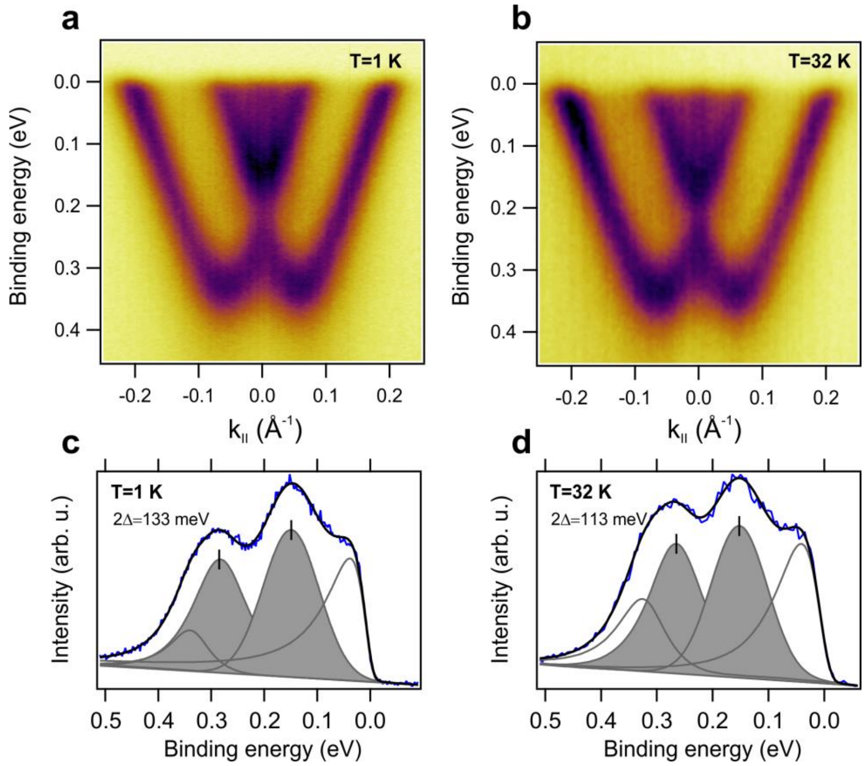

Fig. 5S. (a,b) - ARPES dispersion maps of 3% V-doped BiTeI measured at 1K and 32 K. (c,d) — Corresponding EDCs measured at the Γ point (k_{II} = 0 Å^{-1}) with decomposition into spectral components.

Modification of the experimentally measured KP gap values ΔE at different V-concentrations and temperatures assuming that the energy splitting is of exchange-type and $\Delta E \sim \mu$, where μ is the magnetic moment, would be well described within the framework of the standard power law:

$$\Delta E = E_0(1 - {T}/{T_C})^{1/2}$$

In Table 1 the values of the KP gap for various V-concentrations and at different temperatures are presented. Assuming the critical temperature Tc=130 K, the value of the KP gap at zero-temperature $T = 0\ K$ would be estimated as $\Delta E = E_0$. The KP gap at $T = 0\ K$ presented in the Table 1 (third column) was estimated based on the experimental data in Fig.1 of the main text and using the power law.

**BiTeI+V, %**	$\Delta E\ exp$ (Fig.1), meV	$\Delta E = E_0$ at $T = 0\ K$, meV	ΔE from power law (different temperatures), meV
**0.5%V**	101 (T=20 K)	110	
**2%V**	125 (T=15 K)	130	63,5 (exp = 77 Fig. 4S) at T=99 K
**3%V**	133 (T=1 K)	133,5	116 (exp = 113 Fig. 5S) at T=32 K
**6%V**	105 (T=20 K)	114	

Table 1. Modification of the experimentally measured KP gap values ΔE at different V-concentrations and temperatures within the framework of the standard power law.

3. Estimation of the bulk Curie temperature by the Arrott plots method

In order to characterize the type of phase transitions and to obtain values for the Curie temperature from isothermal magnetization curves *M(H)* we used the modified Arrott plots, which consists in plotting $M^{1/\beta}$ vs. $(H/M)^{1/\gamma}$ [1,2]. In general, Arrott plot is based on Arrott–Noakes equation [1]:

$$(H/M)^{1/\gamma} = \frac{(T - T_C)}{T_1} + \left(\frac{M}{M_1}\right)^{1/\beta}$$

where γ and β are the critical exponents. In the frame of the mean field model, exponents $\beta = 0.5$, $\gamma = 1.0$. According to the mean field model, such curves should reveal a linear behavior in the high field region at different temperatures and the line at *T=Tc* should just pass through the origin. But the mean field model describes a classical collinear ferromagnet. In our case convenient approach based on three-dimensional (3D)-Heisenberg model for ferromagnets with modified critical exponents β=0.365, γ=1.336 was applied [3-7].

Fig. 6S (a,b,c) demonstrates the modified Arrott plots $M^{1/\beta}$ vs. $(H/M)^{1/\gamma}$ according to a 3D-Heisenberg model which are plotted for 2%V, 3%V and 6%V-doped BiTeI.

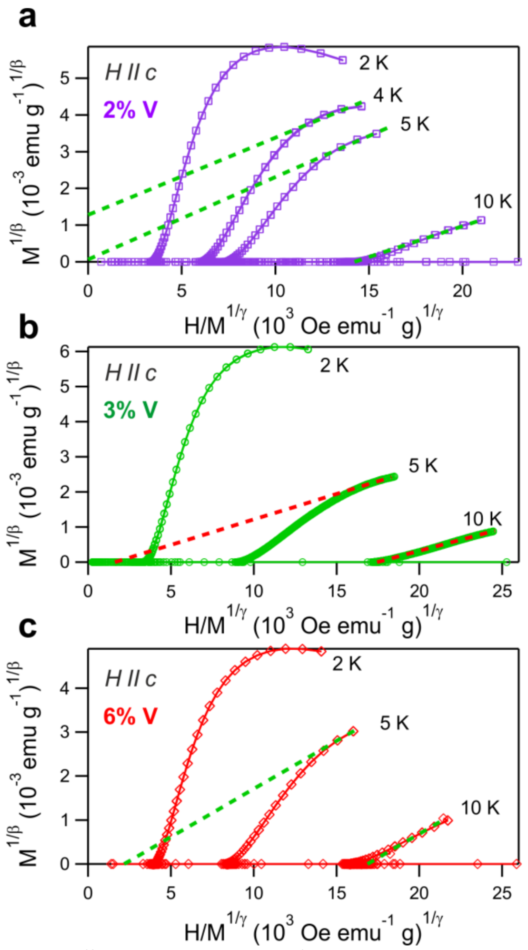

Fig.6S. Modified Arrott plots $M^{1/\beta}$ versus $(H/M)^{1/\gamma}$ obtained from the *M(H)* data in Fig. 3 for 2%V – (a), 3%V – (b) and 6%V-doped BiTeI – (c), plotted by using the 3D-Heisenberg model with critical exponents β=0.365, γ=1.336.

Based on the criterion proposed by Banerjee [8] the magnetic transition is of second order if the Arrott plot curves have a positive slope while the negative slope reflects that the system exhibits a first order transition. The $M^{1/\beta}$ vs. $(H/M)^{1/\gamma}$ curves of our samples exhibit a positive slope and we analyze our data assuming the PM–FM transition to be of second order in this compound according to Banerjee's criteria.

We approximate the $M^{1/\beta}$ vs. $(H/M)^{1/\gamma}$ curves in the high field region in order to obtain the parallel straight lines for critical temperature analysis. To apply the Arrott analysis the straight line crossing the $M^{1/\beta}$ axis corresponds to the temperature higher than the critical temperature, while the straight line crossing the $(H/M)^{1/\gamma}$ axis belongs to the temperature below Tc. And the line passing through the origin corresponds to the Curie temperature. As it is seen in the Fig. 6S, the approximation line of the $M^{1/\beta}$ vs. $(H/M)^{1/\gamma}$ curve for 2%V-doped BiTeI crosses the origin at T=5 K suggesting that the bulk Curie temperature is at ~5 K. For the 3%V and 6%V-doped BiTeI the estimated bulk Curie temperature is slightly below than 5 K.

[1] A. Arrott and J. E. Noakes, Phys. Rev. Lett., 19, 786 (1967)
[2] A. Arrott, Equations of state along the road to Arrott's last plot. J. Magn. Magn. Mat. 322, 1047-1051 (2010)

[3] Kaul, S. N. Static critical phenomenon in ferromagnets with quenched disorder. J. Mag. Magn. Mater. 53, 5–53 (1985)
[4] J. L. Alonso, L. A. Fern´andez, F. Guinea, V. Laliena and V. Mart´ın-Mayor, Nucl. Phys. B, 596, 587 (2001)
[5] M. Ferer, M. A. Moore, and Michael Wortis , Phys. Rev. B 4, 3954 (1971)
[6] Zh. Li, et al. Scientific Reports 10, 15345 (2020)
[7] K. Dhahri et al. RSC Adv., 8, 3099-3107 (2018)
[8] B. K. Banerjee, Phys. Lett., 12, 16 (1964)
